# Understanding the Propensity of ETEC Diarrhea in Endemic Settings—A Review of Epidemiological and Immunological Factors and Vaccine-Related Preventive Measures Using a Multisectoral Approach

**DOI:** 10.3390/microorganisms14091956

**Published:** 2026-09-04

**Authors:** Fahima Chowdhury, Marjahan Akhtar, Firdausi Qadri, Taufiqur Rahman Bhuiyan

**Affiliations:** International Centre for Diarrhoeal Disease Research, Bangladesh (icddr,b), Dhaka 1212, Bangladesh; fchowdhury@icddrb.org (F.C.); marjahan.akhtar@icddrb.org (M.A.); taufiqur@icddrb.org (T.R.B.)

**Keywords:** ETEC, diarrhea, epidemiology, natural immunity, vaccine development

## Abstract

Enterotoxigenic *Escherichia coli* (ETEC) is a leading cause of bacterial diarrheal illness in low- and middle-income countries and among travelers to endemic regions, impacting morbidity and mortality and impairing childhood development. Despite its significant importance in public health, the propensity of ETEC diarrhea in endemic settings is influenced by complex interactions among epidemiological, immunological, environmental, and pathogen-related factors. This review aims to provide a comprehensive understanding of ETEC diarrhea in endemic regions by examining epidemiological trends, host immune responses, environmental determinants, and progress in current ETEC vaccine development. We discuss disease burden, seasonality, age-specific susceptibility, toxin and colonization factor diversity, and their relation to diarrheal disease severity at the community level and in hospitalized patients. In addition, we summarize current knowledge on natural immunity, including mucosal and systemic immune responses induced in ETEC-infected diarrheal patients. The review further highlights advances in ETEC vaccine development, focusing on candidate vaccines, safety, immunogenicity, protective efficacy, and challenges associated with implementation in resource-limited settings. By integrating clinical, microbiological, immunological, environmental, and public health perspectives, this review provides a multisectoral framework to better understand ETEC pathogenesis and prevention. It also highlights future research priorities, vaccine strategies, and policy interventions to reduce the burden of ETEC-associated diarrhea in endemic settings.

## 1. Introduction

*Escherichia coli* is a Gram-negative, facultative bacterium with six recognized diarrhoeagenic pathotypes; among them, enterotoxigenic *Escherichia coli* (ETEC) is the most common pathotype in people living in lower-middle-income countries (LMICs), including travelers [1]. ETEC is the leading bacterial cause of diarrhea in children ≤ 5 years of age and also causes severe dehydrating diarrhea in adults in developing countries due to poor infrastructure, including access to safe water, sanitation, and hygiene (WASH), as well as a lack of health education. Annually, ~220 million of the diarrheal episodes that occur globally are due to ETEC, and there are 75 million ETEC diarrheal cases in children under five, resulting in 18,700–42,000 deaths [2]. Moreover, 11 different study analyses (2011–2016) have shown that ETEC diarrhea is very common in travelers, at around 30–60% among those who visit endemic areas, especially Latin America and Asia [3], and in military personnel from high-income countries deployed to endemic areas. Although ETEC diarrhea among travelers can be self-limiting, antibiotic treatment may work faster at reducing symptoms; unfortunately, traveler’s diarrhea (TD) has become increasingly multidrug-resistant to antibiotics like azithromycin and ciprofloxacin [4]. It has been suggested that the incidence rate for TD is around 10% and self-reported TD is around 30% [5]. Diagnosis is often delayed due to the limited capacity of clinical laboratory diagnostic methods, either for stool culture or polymerase chain reaction (PCR). The actual burden of ETEC diarrhea in developing countries has not yet been established, which has raised some doubts about the reported morbidity and mortality figures. The complex and specialized laboratory tests required to detect ETEC in these poor endemic settings have been a limiting factor. Studies have shown that non-severe ETEC diarrhea and asymptomatic infections have a detrimental effect on gut enteropathy and have a long-term impact on child growth and cognitive development [6]. In addition, extensive use of antibiotics leads to antimicrobial resistance in ETEC strains. Estimated data from a meta-analysis suggest an additional 89,000 deaths/year occur in Africa and South Asia in children above 5 years of age [7]. Bangladesh is an endemic country for ETEC diarrhea, where it is the second most common bacterial cause after *Vibrio cholerae* O1 (*V. cholerae* O1) [8]. More studies must be conducted in this community to assess the risk and actual numbers of ETEC cases above 5 years of age to control ETEC in this population as well. A retrospective analysis of an oral cholera vaccine clinical trial in an urban slum, Dhaka (2011–2015), showed that the ETEC incidence was 150/100,000 person-years (PY) in the community, which increased in people over 45 years old (219/100,000 PY); the highest rate was seen in children less than 1 years old (2007/100,000 PY) [9].

## 2. Epidemiology and Pathogenesis of ETEC

### 2.1. Transmission

Humans are primarily a source of ETEC infection and spread disease through the fecal oral route. Fecal contamination in environmental sources like drinking water, surface water, soil, and crops further spreads the disease. ETEC has been associated with diarrhea in pigs, calves, rabbits, and domestic animals and may also be a source of disease transmission in households in LMICs [10,11].

Heat-stable enterotoxins (STa, STp, and STh) are common in ETEC found from humans and livestock; however, STb is the only enterotoxin found in livestock ETEC [12]. Recent studies have shown that weaned piglets are at risk for post-weaning diarrhea (PWD) [13]. The F17 strains are responsible for diseases in calves and other livestock animals like lambs and alpacas [14]. The IgA antibody is strongly associated with protection against ETEC diarrhea in animals [15]. A multivalent ETEC vaccine is required to protect animals from PWD. Recently, two oral live ETEC vaccines, Coliprotec^®^F4 and Coliprotec^®^F4/F18, were recommended for protection against PWD. Hence, we need to focus on new vaccines that include LT, STb and/or STa, and F4 [15], and in the case of a multidrug-resistant ESBL-/pAmpC-producing ETEC pathogen, diarrheic foals can act as a potential reservoir [10].

### 2.2. Virulence Factors and Host Susceptibility

ETEC causes a wide spectrum of clinical manifestations, including asymptomatic, mild, moderate, and severe diarrhea, through two key virulence factors: colonization factors (CFs) and enterotoxins. ETEC pathogens secrete heat-labile toxin (LT) and/or heat-stable toxins (STh and STp). There are over thirty types of colonization factors (CFs) of ETEC, the most common of which are CFA/I, CS1-7, CS14, CS17, and CS21 [1,16].

### 2.3. Seasonality of ETEC Diarrhea

In Bangladesh, ETEC diarrhea is common throughout the year, but a biannual seasonal peak has been observed; it first appears in the summer season, when bacterial growth is profound in the environment, and reappears during monsoon or rainy season due to the heavy contamination of surface water with fecal material—via global climate change—thereby enhancing disease spread [17,18]. Moreover, studies in Egypt have shown that toxin-producing phenotypes vary by season; the ST ETEC strain is common in summer, whereas LT-producing toxin ETEC strains exist throughout the year and do not show any seasonal pattern [19,20]. Policy makers’ understanding of the seasonal pattern distribution of ETEC will help their implementation of precautionary measures, such as vaccination programs, sanitation procedures, and food safety measures. In Mexico, a lower attack rate is observed among travelers during winter months, and the rate increases in hot summer; the ETEC infection rate increases by 7% for each degree centigrade [21]. In Taiwan, seasonal variation has also been observed in aquatic environments in spring and summer [22].

### 2.4. Clinical Manifestation

The severity of ETEC diarrhea is very similar to that of *V. cholerae*. Symptoms begin within 1–3 days after the ingestion of contaminated food or water and sometimes persist up to 7 days. The pathogen causes profuse fluid secretion from the small intestine and features frequent, loose, non-bloody, watery stools without mucus or fecal leukocytes. Other common features include moderate-to-severe abdominal cramps and bloating. Patients are generally afebrile, or have a low grade of fever. Nausea is commonly reported, as well as vomiting and varying degrees of dehydration. Electrolyte imbalance is also common in dehydrated or malnourished children without fluid replacement [23]. Asymptomatic or recurrent infection of ETEC diarrhea leads to protein loss in the gut, which induces gut enteropathy and leads to severe malnutrition, as well as stunting, irritable bowel syndrome (IBS), and a compromised immune system. Ultimately, this results in growth retardation and diminished cognitive development [24]. Blood groups ABO, including A and AB, are risk factors for developing severe ETEC diarrhea [25].

### 2.5. Global Burden

The GEMS study (Global Enteric Multicenter Study) showed that medically attended moderate-to-severe diarrhea (MSD) in children less than 5 years old is associated with a higher case fatality rate (CFR) compared to those without diarrhea due to ETEC. Elderly individuals also had a higher chance of mortality [26]. Moreover, irritable bowel syndrome is very common in TD (~10–14%) due to the ETEC pathogen [24]. In Africa and Asia, ETEC had the highest median prevalence, around 22%. The MAL-ED study showed that persistent diarrhea is more common in infants and children under 5 years of age due to ETEC in South America and is also responsible for TD in countries such as Asia, Mexico, and Africa, as well as in Central and South America [2]. In Asia, the highest number of ETEC diarrheal deaths was recorded across all age groups, while individuals over 5 years old experience most of these deaths in Africa and South Asia. In contrast, in eastern sub-Saharan Africa, it is those under 5 years of age who experience most of these deaths (~5485 deaths). The worldwide ETEC mortality rate ranges from 0.1 to 8.8/100,000 [27,28].

## 3. Age-Related ETEC Infections and Severity in Children and Adults

### 3.1. Incidence in Young Children

Over the years, several studies conducted on the ETEC pathogen have revealed a significant and frequent cause of bacterial diarrhea in younger children less than 2 years of age [19,29]. In Egypt, studies have shown that 70% of the first episodes of diarrhea in infants occur due to the ETEC pathogen, where the incidence rate is higher in male than female children [19]. Another birth cohort study in Peru showed that the ETEC incidence rate was 73 per 100 child-years under an age of two. A community-based study in India showed 20% prevalence for those under 2 years of age [30]. Moreover, in Bangladesh, 90% of ETEC diarrheal cases who visited the hospital were children of 3 months to 2 years of age [29], and in a birth cohort study, ETEC was found to be the most common cause of diarrhea in children 0 to 2 years of age, accounting for around 18% of all diarrhea types [31]. Different community-based studies in West Africa, Egypt, and Bangladesh and hospital-based surveillance data from Bangladesh show that the rate of ETEC infections increases by 3 to 6 months of age [19,32]. Recently, a community-based trial was conducted, revealing incidence rates of 48% and 5% for children under 5 years of age and from 5 to 15 years of age, respectively.

### 3.2. Adult Disease Burden

A limited amount of age-related incidence data are available for adults, mostly from India and Bangladesh. A community-based trial in Bangladesh showed that ETEC diarrhea is prevalent in adults by 47% more than those 15 years old. Hospital surveillance data from two rural coastal areas, Chhatak and Mathbaria, in the northern and southern parts of Bangladesh revealed that ETEC was high in adults between 20 and 60 years of age and required hospital care [33]. The incidence of ETEC diarrhea differs by country, including Bangladesh, Mexico, Peru, Egypt, Argentina, Nicaragua, and Tunisia, and global studies have highlighted significant geographical variation in ETEC incidence among symptomatic and asymptomatic children [34,35,36], where environmental and immunological factors significantly contribute to these observed changes in disease incidence. Improved WASH (water, sanitation, and hygiene) infrastructure, safe drinking water, and effective waste disposal are main contributing factors in the LMIC. Moreover, herd immunity in endemic settings, nutritional status, socioeconomic conditions, and methodological differences in study designs play important roles.

### 3.3. Age-Related Severity

In these endemic settings, hospital-based diarrhea resembling severe cholera has been shown in adults with ETEC infection, and they experienced more severe dehydration than children and infants [16]. Interestingly, elderly patients who are more than 65 years old presented with more severe dehydration than children due to ETEC infections [37]. A very recent publication on community-based ETEC diarrhea showed that only 2.6% of participants below 5 years of age had severe dehydration, while 75.4% showed no signs of dehydration. Severe dehydration was presented by 20.8% of patients 5–14 years of age and by 35.2% of patients above 15 years of age (Table 1). A significant association was identified between age at presentation and the dehydration status of ETEC diarrheal cases [9]. These data reveal that the incidence of ETEC diarrhea was also higher in children under 5 years of age and adults, but severe dehydrating diarrhea was higher in adults and elderly people. The incidence in younger and older populations may be due to the lower level of natural ETEC immunity in both age groups and the gradually declining antibody response in older people [1]. Severe ETEC diarrhea in adults may be due to age-related healthcare-seeking behaviors, as parents in this setting are quick to seek care for their children if they develop diarrhea. Moreover, parents begin administering ORS to their children as soon they develop diarrhea at home.

### 3.4. Distribution of ETEC Toxin in Different Age Groups

ETEC toxins significantly vary across different age groups. A recent community trial conducted in Dhaka revealed that children under 5 years of age and children 5–14 years old suffer more from LT ETEC diarrhea. However, the GEMS study showed that children under 5 are vulnerable to LT-ST- and ST-toxin-type ETEC diarrhea [33]. Variation in toxin type and the predominance of a specific toxin in ETEC diarrhea can vary across different age groups as well as geographical locations and may be influenced by factors such as genetic coding and infectious doses. In addition, variations in toxin expression among ETEC strains may contribute to differences in clinical severity. Our findings show that LT-only ETEC was less commonly associated with severe dehydration, which is supported by previous reports [9]. Overall, these observations highlight the importance of designing future ETEC vaccines with broad coverage to target the diversity of circulating ETEC strains. However, toxin-positive, CF-negative ETECs are frequently detected in epidemiological studies [38]. Porcine and human ETEC have similar virulence factors (heat-stable enterotoxins STp and STh) and type II secretory system (T2SS) protein YghG, responsible for the secretion of LT. These are pathogenic, easily transmitted and adapted to the human host [39,40]. Hence, cross-transmission occurs between animals and humans due to a shared mechanism.

## 4. ETEC Infection and Malnutrition

The Malnutrition and Enteric Disease birth cohort study (from birth to 24 months of age), conducted on eight distinct geographic locations, showed that ETEC infections per 100 child-months were higher in Tanzania (54.81) and Bangladesh (46.75) and that anthropometric failure was significantly associated with asymptomatic ETEC infection in the Bangladesh, India, and Tanzania sites [41]. A birth cohort study performed in Bangladesh showed that a higher percentage of children with ETEC diarrhea were underweight and stunted compared to children with non-ETEC diarrhea by 12 months, and stunting persisted up to 24 months (45% vs. 31%) [31]. ST-toxin ETEC diarrhea among children 12–23 months old with moderate-to-severe dehydration showed significant association with acute malnutrition, compared to well-nourished children.

## 5. Immune Responses Against Natural ETEC Infection

### 5.1. B Cell and Antibody Responses

ETEC-infected diarrheal patients develop robust mucosal IgA antibodies specific to LT and CFs. Secretory IgA (SIgA) has been found to play a role in the clearance of ETEC bacteria [42]. Because ETEC does not invade intestinal epithelial cells, it is believed that immune protection is most likely mediated by antigen-specific antibodies, particularly locally produced SIgA antibodies on the mucosal surface, which can inhibit bacterial adhesion, neutralize enterotoxins, and prevent colonization [42,43]. Despite this biological rationale, the direct protective role of SIgA against ETEC diarrhea in humans has not been conclusively demonstrated, and no validated immunological correlate of protection (CoP) has been established yet. However, one study has shown that orally administered monoclonal SIgA antibodies targeting the ETEC colonization factor CfaE significantly reduced intestinal colonization in a mouse challenge model [44]. A treatment comprising 10^7^ CFU of ETEC with 10 mg/kg of SIgA1 or SIgA2 monoclonal antibodies resulted in an approximately 10–100-fold reduction in ETEC colonization compared with controls, providing strong experimental evidence that SIgA can effectively limit ETEC colonization at the mucosal surface. In contrast, although numerous controlled human infection model (CHIM) studies have been conducted to investigate immune responses following ETEC challenge, most have focused on evaluating systemic or surrogate mucosal antibody responses, including antigen-specific IgA levels measured in serum, ALS, saliva, or fecal extracts, rather than directly quantifying pathogen-specific SIgA [45]. Measuring SIgA in stool remains technically challenging due to variable stool consistency, uneven antibody distribution, dietary influences, proteolytic degradation, and the lack of standardized collection, processing, and normalization methods [46]. Hence, the development of standardized, sensitive, and reproducible assays for quantifying antigen-specific SIgA in stool and other mucosal specimens is a critical research priority. Such assays, combined with well-designed longitudinal cohort studies and CHIM investigations, would facilitate rigorous evaluations of SIgA as a potential immunological correlate of protection and accelerate the development and evaluation of effective ETEC vaccines.

ETEC infection induces both systemic and mucosal antibody responses in diarrheal patients. Different studies conducted on LT-positive ETEC-infected patients have shown increased antibody levels in the acute phase of infection, which dropped to healthy control levels by the convalescent period on day 30 [47]. However, LT-specific memory B cell responses were induced on day 30, and since anti-LT immunity gives relatively short-term protection in humans, reinfection is common with LT-expressing ETEC [31,48].

Studies indicate that immunity against ETEC CFs probably provides long-lasting immunity to ETEC [49,50]. Our studies show that reinfections with ETEC-expressing homologous CFs were not common in children who had a primary infection with CFA/I, CS1+CS3, CS2+CS3 or CS5+CS6 expressing ETEC strains [31]. This finding suggests that primary ETEC infection with these antigens enhance protective immunity against self- and cross-reacting epitopes. This cross-protection against heterologous CFs depends on the natural priming status, as Bangladeshi adults, but not North American volunteers, infected with CFA/I strains responded to both homologous and heterologous CF antigens [51]. In line with this, increased levels of CF-specific mucosal and systemic antibody responses were found in ETEC-infected diarrheal patients up to one month after infection [52]. ETEC infection has also demonstrated a promising systemic antibody response against both EtpA and EatA [53,54]. Similarly, the evaluation of antibody-in-lymphocyte supernatant (ALS) specimens from ETEC-challenged human volunteers indicated promising responses to both antigens [55,56].

### 5.2. T Cell and Cytokine Responses

T-cell-mediated immune responses play an important complementary role in host defense and immune memory by inducing antibody-mediated immunity. Naturally exposed ETEC diarrheal patients have demonstrated induced memory CD4+CD45RO+Th cells with a Th17 phenotype (CCR6+CXCR3-) in freshly isolated peripheral blood mononuclear cells (PBMCs) [52]. Human experimental challenge studies have also demonstrated that ETEC infection induces antigen-specific CD4^+^ T cell responses, with a predominant skew toward Th1 and Th17 phenotypes. The induction of ETEC-specific Th17 responses was further supported by the increased levels of LTB- and CS6-specific IL-17A secretion from PBMCs. Th17 cells and their secreted IL-17A cytokines are particularly important in noninvasive infection due to their involvement in mediating protective immunity. In addition to ETEC, Th17 cells also provide protection against other bacterial infections in the intestinal tract by inducing inflammation through neutrophil recruitment, helping in antimicrobial peptide production, or enhancing the development and secretion of IgA over the mucosal epithelium [57,58,59]. Our study shows a correlation between IL-17A and mucosal IgA responses found in ETEC-infected diarrheal patients, suggesting that Th17 responses play a role in promoting the development of mucosal B cell responses [52]. In parallel, Th1 responses characterized by IFN-γ production may support macrophage activation and contribute to bacterial clearance. Notably, differences in the ETEC-specific cytokine production and gut-homing capacity of CD4^+^ T cells were observed, with increased expression of the mucosal homing integrin α4β7 on circulatory T follicular helper (Tfh) cells correlating with reduced stool volume and enhanced ETEC-specific IgA memory B cell responses. A study on ETEC vaccination also found significantly increased levels of activated ICOS+ CXCR5+circulatory Tfh cells [60]. Overall, these findings support the role of CD4^+^ T cells, particularly Tfh cells with gut-homing potential, in shaping both clinical outcomes and the development of protective mucosal humoral immunity following ETEC exposure. Future studies should focus on defining antigen-specific CD4^+^ T cell subsets, including Th17 and T follicular helper cells, that correlate with protection and reduced disease severity following ETEC infection. Identifying durable, mucosal-homing T cell responses and their role in supporting protective antibody immunity will be critical for establishing cellular correlates of protection and guiding the rational design and evaluation of ETEC vaccines in pipeline.

## 6. ETEC Vaccines Under Clinical Development

Evidence from different clinical and field studies shows that natural infection or human challenge with the ETEC pathogen provides protective immunity [2,61]. Community studies have shown that ETEC infection declines after the age of 5 until about 10 years. ETEC can be prevented when infection is caused by the same ETEC phenotype. Colonization factors, adhesins, and LT are the main drivers of protection against ETEC diarrhea. Studies conducted in various geographical locations under LMIC settings have focused on genotyping the ETEC pathogen and have revealed that CS6, CFA/I, CS3, and CS5 are the most common colonization factors, causing ~ 90% ETEC diarrhea. In addition, more recently, CS21 has been increasingly detected in patients with diarrhea [62]. Understanding phenotypic factors is important for developing a protective ETEC vaccine by including broad and antigenic coverage. Current strategies for ETEC vaccines under clinical development are shown in Table 2.

To maximize the immunogenicity and protective efficacy of the vaccine, both anti-toxigenic and anti-colonization properties need to be induced during vaccine development. Recently, adjuvants have been added to the vaccine formulation to increase vaccine efficacy. Coadministration of enteric vaccines can be beneficial for children requiring them. In endemic settings, bacterial pathogens such as ETEC and *V. cholerae* or ETEC and rotavirus are common. Studies have shown that ETEC vaccines requirements are different for adults and pediatric populations. Thus, some clinical considerations should be focused on while developing ETEC vaccines for the pediatric population: gut enteropathy, malnutrition, coinfection, and microbiota. A major gap remains in our understanding of the incidence of ETEC diarrhea, as no community studies have been conducted on the health-seeking behavior of those prone to infection.

### 6.1. ETVAX

The most advanced ETEC vaccine in development is the second-generation whole-cell oral ETEC vaccine ETVAX, which consists of inactivated *E. coli* strains over-expressing CFA/I, CS3, CS5, CS6, and LT-like toxoid LCTB, as well as the mucosal adjuvant double-mutant LT (dmLT) [63]. Phase I studies were carried out in Sweden with different prototypes of the ETVAX vaccine [64], found to be safe and immunogenic. Then, Phase I/II trials were carried out in adults and children of various ages, including infants [65,66]. The vaccine showed excellent safety in adults as well as both systemic and mucosal immunogenicity against all five of the key vaccine antigens [65], and adverse events such as vomiting were observed in the youngest age group with lower immunological responses [66]. However, dmLT was shown to enhance the strength and breadth of immune responses in infants [67]. A double-blind Phase-2b study was conducted in 750 Finnish travelers to Benin, West Africa [68]. The ETVAX vaccine induced significant protection against moderate-to-severe ETEC-attributable disease. It was also tested in a large Phase 2b trial evaluating the safety, immunogenicity, and efficacy of ETVAX in 6–18-month-old children in The Gambia [69]. Vaccine efficacy was measured against moderate-to-severe ETEC diarrhea (MSD-ETEC); the vaccine was safe, serious adverse events were rare, and increased immune responses were observed against the vaccine antigens. Vaccine efficacy against the primary endpoint was 26.6%. In secondary analyses, efficacy against MSD-ETEC irrespective of co-pathogens was 48.2%, and it increased to 80.6% when enteroparasitic co-pathogens were excluded. Among children vaccinated before 9 months of age, efficacy against all MSD-ETEC regardless of co-pathogens was 67.8%. Additionally, vaccine efficacy against moderate-to-severe diarrhea of any cause was 21.4% [69]. Based on the promising clinical performance of the ETVAX vaccine, future Phase III efficacy trials should be conducted across diverse LMIC settings and with different age groups to further evaluate its protective efficacy and generalizability.

### 6.2. ACE527

ACE527 is a live-attenuated oral ETEC vaccine that aims to mimic natural infection and induce robust mucosal immunity. It ACE527 comprises three strains engineered to express CFA/I, CS1, CS2, CS3, CS5, CS6, and LTB. The enterotoxin and antibiotic resistance genes are deleted, and knockout mutations are introduced into the aroC, ompC, and ompF genes to attenuate the strains. This vaccine was found to be well tolerated at doses of up to 10^11^ CFUs and elicit significant immune responses against colonization factors [70]. However, in a subsequent Phase IIb study evaluating two doses of 10^11^ CFU, only 27% protective efficacy against moderate-to-severe diarrhea was observed. Additionally, this vaccine induced significantly more vomiting compared to in the placebo group [71]. Due to its limited efficacy, further development of the vaccine was discontinued.

### 6.3. Fimbrial Tip Adhesin Vaccine

Fimbrial tip adhesin is a subunit ETEC vaccine, developed based on CFA/I, a well-characterized CF antigen, and fimbrial tip adhesin CfaE is the minor subunit located at the distal end of the CFA/I pilus, representing a rational, mechanism-based vaccine target ETEC as it mediates the critical initial attachment of bacteria to the intestinal epithelium [72]. In a Phase I clinical trial, the transcutaneous delivery of a donor strand-complemented CfaE (dscCfaE) construct demonstrated safety but limited immunogenicity, prompting the evaluation of alternative formulations and routes. The protective efficacy of the vaccine was evaluated following intradermal administration of dscCfaE and a chimeric construct in which dscCfaE replaced the A1 domain of cholera toxin and assembled with heat-labile toxin B subunits [73]. These formulations were co-administered with the mutant LT adjuvant LTR192G (mLT) to enhance immunogenicity. Both prototypes were well tolerated, inducing predominantly mild local reactions, and elicited robust serum and antibody-secreting cell responses, particularly with the higher 25 µg dose of dscCfaE plus mLT [73]. In a controlled human infection model (CHIM) using the homologous CFA/I^+^ ETEC strain H10407, three intradermal doses of 25 µg CfaE with 100 ng LTR192G conferred an overall vaccine efficacy of 27.8% against moderate-to-severe diarrhea, while significantly reducing stool output and overall disease severity [74]. These findings provide the first demonstration of the protective efficacy of an intradermally delivered ETEC adhesin vaccine in humans and support the continued development of CfaE-based strategies as components of multivalent ETEC vaccine platforms.

### 6.4. dmLT

dmLT is widely recognized as a potent mucosal adjuvant; however, it has also been developed as a standalone vaccine candidate against ETEC [75]. This vaccine has been evaluated in several Phase 1 clinical trials under endemic and non-endemic settings to determine its safety, optimal dose, and route. Studies have revealed that both the dose and route of administration critically influence the magnitude of immune responses as well as the safety profile against the dmLT vaccine. In a Phase I clinical trial conducted in 36 US adults, a single dose of the vaccine was administered in escalating doses of 5 µg, 25 µg, 50 µg, and 100 µg, which were found to be safe and well tolerated. Specifically, 50 µg of dmLT exhibited the highest serum and fecal antibody responses [76]. In contrast, the sublingual delivery of three doses of the dmLT vaccine in 80 US adults induced moderate immune responses [77]. Recently, in a Phase I, double-blind, dose-escalation study (0.1–2.0 μg), three doses of the dmLT vaccine were administered intradermally at three-week intervals and were shown to be well tolerated [78]. However, participants experienced mild local reactions, occasional gastrointestinal adverse events, and frequent hyperpigmentation at the injection site. Intradermal vaccination induced robust immune responses, including elevated serum IgG and IgA antibodies, LT-neutralizing antibodies, and IgG and IgA antibodies in lymphocyte culture supernatants. The strongest immune responses were observed at the highest dose level (2.0 μg) [78].

A Phase I randomized, placebo-controlled study conducted in Bangladesh further evaluated the safety and immunogenicity of the dmLT vaccine administered orally, sublingually, and intradermally [79]. Participants received either three oral or sublingual doses of 5 μg or 25 μg dmLT administered at two-week intervals or two intradermal doses of 0.3 μg dmLT administered three weeks apart. Overall, dmLT was well tolerated across all administration routes, with few, mild, and transient adverse events reported. The strongest immune responses, including serum, ALS, and ASC IgA and IgG responses as well as LT-neutralizing antibodies, were observed among recipients of the 25 μg oral dose and the 0.3 μg intradermal dose. In contrast, similarly to in US adults, sublingual administration induced comparatively modest immune responses, with minimal serum antibody responses following the 5 μg dose, and only sporadic ALS and ASC responses were observed with both the 5 μg and 25 μg doses [79]. All data from Phase I studies indicate that both antigen dose and route critically influence immunity magnitude, with oral (25–50 μg) and intradermal (0.3–2.0 μg) routes demonstrating superior immunogenicity compared with sublingual delivery.

### 6.5. ShigETEC Vaccine

ShigETEC is a combined vaccine candidate designed to provide protection against both shigellosis and ETEC diarrhea [80]. This vaccine is based on a live-attenuated, noninvasive Shigella flexneri 2a strain (2457T), which has been genetically modified through the deletion of the rfbF and setBA genes as well as the invasion plasmid antigen genes ipaB and ipaC. The engineered shigella strain was further designed to express an ETEC fusion antigen consisting of the LTB and a detoxified form of the STa toxoid. In a Phase I trial in Hungary, oral immunization of ShigETEC was well tolerated and safe up to four doses with 5 × 10^10^ CFUs. This vaccine showed strong immunogenicity, particularly IgA antibody responses against ShigETEC lysate antigens [81]. In contrast, immune responses against LTB were much weaker, and no significant STa IgG or IgA response has been detected from any of the vaccinees. A double-blind, randomized, placebo-controlled, dose-escalation Phase I study on this ShigETEC vaccine is ongoing in Bangladesh in adults and children of different age groups. The results from this study will provide new insights into the ShigETEC vaccine’s immunogenicity in different age groups.

### 6.6. CVD 1208S-122

CVD 1208S-122 is an oral live-attenuated bivalent vaccine against Shigella and ETEC that consists of an attenuated S. flexneri 2a strain expressing the ETEC CFA/1 and A2 domain and B subunit of LT [82]. A recent Phase 1 safety and immunogenicity study on CVD 1208S-122 demonstrated that the vaccine has no safety concerns and that the vaccine induced antibody responses against S. flexneri 2a LPS and ETEC antigens CFA/I and LT [83].

## 7. Conclusions

Overall, understanding the propensity of ETEC diarrhea in current times requires comprehensive evaluation of epidemiological patterns, host immunological responses, and effective preventive strategies. Epidemiological studies have been conducted in Bangladesh that help identify high-risk populations, seasonal trends, and transmission dynamics under endemic settings. At the same time, investigations into immunological factors such as mucosal immunity, antibody responses, and memory B cell development have provided insight into natural protection and susceptibility to infection. Integrating these findings with vaccine-related preventive measures, including the development and evaluation of candidate ETEC vaccines, is essential for long-term disease control. A multisectoral approach that combines public health surveillance, laboratory-based immunological research, vaccine development, and community-level interventions can significantly improve approaches with the aim of reducing the burden of ETEC diarrhea in Bangladesh and globally.

## Figures and Tables

**Table 1:** Age-related ETEC infections and severity in children and adults.

**Incidence of ETEC and severe ETEC diarrheal episodes, from a cluster-randomized control trial in an urban slum, Dhaka (2011–2015)** [9]										
Age Groups	Number	ETEC Cases		Incidence rate/100,000 person-years (95% CI)		*p*	Cases	Severe Dehydration IR (95% CI)		*p*
Overall	408,706	1041		150 (141, 159)		<0.001	196	28 (24, 32)		<0.001
<1 year	20,728	176		1353 (1164, 1564)			0	0 (0, 19)		
1–4 years	44,919	324		621 (556, 692)			13	25 (14, 41)		
5–14 years	85,115	53		38 (29, 50)			11	8 (4, 14)		
15–45 years	265,744	330		80 (72, 89)			109	26 (22, 32)		
>45 years	41,651	158		196 (168, 229)			63	78 (61, 100)		
**Comparison of adults and children hospitalized with ETEC diarrhea, in Dhaka, Bangladesh, from 1996 to 2002** [1]										
			Adult ETEC (15–80 years; median 30 years)		Children ETEC (0.1 to 36 months; median 0.83 years)				*p*	
ETEC			478		1107					
Severe Dehydration			36%		3%				<0.001	
Moderate Dehydration			41%		24%					
Mild Dehydration			23%		73%					
2% hospital surveillance data are from 15,558 patients seen at the diarrhea hospital in Bangladesh based on routine surveillance for enteric pathogens										
**Incidence of ETEC and severe ETEC diarrheal episodes, from an epidemiological study in rural areas of Bangladesh from 2014 to 2015** [33]										
			ETEC cases		Severe dehydration				Some Dehydration	
<5 years			28%		0%				5%	
5–20			18%		5%				3%	
20–60			45%		12%				18%	
>60 years			7%		5%				0%	

**Table 2:** Status of ETEC vaccine development.

Name of the Vaccine	Preclinical	Phase I	Phase IIA	Phase IIB	Phase III
MecVax	Oral, protein-based vaccine, University of Illinois Urbana-Champaign				
EtpA adhesin	Oral, sublingual, and intradermal, EtpA with dmLT, Washington University School of Medicine				
ACE527			Oral, live-attenuated vaccine combined with dmLT, PATH		
ETVAX				Oral, inactivated vaccine, Scandinavian BioPharma, SBH	
ShigETEC		Oral, live-attenuated vaccines, Eveliqure Biotechnologies GmbH			
CVD 1208S-122		Oral, live-attenuated vaccines, Center for Vaccine Development and Global Health, University of Maryland			
Fimbrial tip adhesins (FTA)			Oral, sublingual, intranasal, and intradermal, subunit, recombinant, polysaccharide, and conjugate vaccines, CFA/I tip adhesin fusion proteins along with dmLT, Naval Medical Research Center		
dmLT		Oral, sublingual, intradermal, Tulane University School of Medicine			

## Data Availability

No new data were created or analyzed in this study. Data sharing is not applicable to this article.
